# Development and validation of a questionnaire to identify severe maternal morbidity in epidemiological surveys

**DOI:** 10.1186/1742-4755-7-16

**Published:** 2010-07-21

**Authors:** Joao P Souza, Jose G Cecatti, Rodolfo C Pacagnella, Thaís M Giavarotti, Mary A Parpinelli, Rodrigo S Camargo, Maria H Sousa

**Affiliations:** 1 Department of Obstetrics and Gynecology, School of Medical Sciences, University of Campinas-UNICAMP, PO Box 6030, 13083-881 Campinas-SP, Brazil; 2 Center of Studies in Reproductive Health of Campinas-CEMICAMP, Campinas, Brazil

## Abstract

**Objective:**

to develop and validate a questionnaire on severe maternal morbidity and to evaluate the maternal recall of complications related to pregnancy and childbirth. *Design: *validity of a questionnaire as diagnostic instrument. *Setting: *a third level referral maternity in Campinas, Brazil. *Population: *386 survivors of severe maternal complications and 123 women that delivered without major complications between 2002 and 2007.

**Methods:**

eligible women were traced and interviewed by telephone on the occurrence of obstetric complications and events related to their treatment. Their answers were compared with their medical records as gold standard. Sensitivity, specificity and likelihood ratios plus their correspondent 95% confidence intervals were used as main estimators of accuracy. *Main outcomes: *diagnosis of severe maternal morbidity associated with past pregnancies, including hemorrhage, eclampsia, infections, jaundice and related procedures (hysterectomy, admission to ICU, blood transfusion, laparotomy, inter-hospital transfer, mechanical ventilation and post partum stay above seven days).

**Results:**

Women did not recall accurately the occurrence of obstetric complications, especially hemorrhage and infection. The likelihood ratios were < 5 for hemorrhage and infection, while for eclampsia it almost reached 10. The information recalled by women regarding hysterectomy, intensive care unit admission and blood transfusion were found to be highly correlated with finding evidence of the event in the medical records (likelihood ratios ranging from 12.7-240). The higher length of time between delivery and interview was associated with poor recall.

**Conclusion:**

Process indicators are better recalled by women than obstetric complication and should be considered when applying a questionnaire on severe maternal morbidity.

## Background

Each year, more than 500,000 avoidable maternal deaths occur worldwide, the majority in the developing world [1]. Alongside family planning and preventing unsafe abortions, the most effective actions for the reduction of maternal mortality are those implemented immediately following the onset of an unexpected complication during pregnancy or childbirth. Delays in implementing required interventions have been associated with the inequality in maternal mortality between developed and developing countries [2].

These delays in health care provision can be identified by auditing the cases of survivors of severe and acute complications [3]. However, as surveillance of severe maternal morbidity (near miss) is not a rule, the use of population health surveys could be an alternative to obtain information on the barriers that women had to overcome to receive adequate obstetric care [4].

For many years, demographic and health surveys (DHS) have been used to study maternal and perinatal heath in developing countries [5]. A systematic review has observed that population surveys using validated questionnaires provided useful information on the prevalence of maternal morbidities [6]. However few nationwide population surveys have used formally validated questionnaires. Prior validation of questionnaires on maternal morbidity would be advised to improve the quality of the information, once large variations were observed among different obstetric complications.

Estimating the prevalence of severe maternal morbidity and evaluating various associated factors can be useful to improve health systems. Thus, we developed a questionnaire on severe maternal morbidity, as a tool to identify the survivors of severe and acute complications related to pregnancy to be used in the Brazilian Demographic Health Survey [7] and probably also in other studies using similar approaches. It is expected to provide valuable information on the occurrence of maternal complications and the need for especial procedures for the care of women during pregnancy and childbirth as a proxy for identifying acute episodes of severe maternal morbidity or maternal near miss. Therefore, the aim of this study was to develop and validate this questionnaire and to evaluate the maternal recall of complications and procedures for the care related to pregnancy and childbirth.

## Materials and methods

This study presents the development of a questionnaire to investigate severe complications during pregnancy by maternal report and assesses its validity. It was carried out at a tertiary teaching maternity hospital, after approved by the Institutional Review Board.

A pre-coded, structured questionnaire to investigate severe maternal morbidity was built based on previous validated questionnaires [4,8-13]. The aim of this questionnaire was to identify the most severe cases of maternal morbidity by women recall and for that, questions related to direct obstetric complications contributing to maternal deaths were included (pre-eclampsia/eclampsia, haemorrhage, infection and jaundice) [14]. Questions on selected process indicators were also included as proxies of severe maternal morbidity (admission to ICU, blood transfusion, hysterectomy, transfer to a referral hospital, laparotomy, etc.). They have been previously used to identify severely ill women during pregnancy and childbirth [15]. This questionnaire was developed in Brazilian Portuguese, and was pre-tested in an independent sample of women through telephone interviews. Minor refinements were performed after pre-testing. The complete content of the questionnaire is shown in Additional file 1. The validity of this questionnaire was assessed using a case control design approach.

For selection purposes we have defined cases as women who had been admitted to intensive care unit (ICU) of the institution between October 2002 and September 2007 and had any event of severe maternal complications as pre-eclampsia/eclampsia, hemorrhage, infection, jaundice or had being submitted to any procedures regarding treatment for a maternal complication, as hysterectomy, blood transfusion, laparotomy, inter-hospital transfer, mechanical ventilation or postpartum stay longer than one week. Controls were defined as women who did not present any severe complication or did not have any procedure as above and were identified among those staying in the rooming-in ward after delivery in the same period. We arbitrarily defined a proportion of 3 cases to 1 control and the selection process was performed choosing by random the first woman with hospital discharge in the same day each third case with severe maternal morbidity had been also discharged. Information on their medical condition is well defined and clearly described in the clinical records using standard forms, considered of good quality and with no missing information, thus decreasing the likelihood of selection bias.

Eligible subjects were identified retrospectively according to the hospital information system that provided a list containing detailed contact information for them. Considering the need for interviewing women at different time periods after the childbirth and also that all eligible women had their telephone contacts (either a fixed or cell phone number) informed in the medical records at the moment of hospital admission, we took the decision to perform the interview through a CATI (Computer Assisted Telephone Interview).

During the period from July through October 2007, the eligible women were contacted by telephone for verbal consent and to schedule the interview. Three trained female interviewers contacted the women through telephone, under the supervision of a research assistant skilled in reproductive health teleresearch. Multiple efforts were made to trace the majority of them, however the protocol established that those women who were unable to be traced via telephone after 5 unsuccessful attempts were excluded. Once contacted and the women consent to participate in the study, the protocol allowed as many attempts as necessary to obtain the interview. For the interview, a mixed list including only name, medical register and contact information of women eligible as case or control was prepared by the principal investigator to maintain the interviewers unaware of the actual condition of each woman in this regard. The list was reviewed every week and a new one was released in order to keep the proportion of cases and controls.

The interviews were recorded and the answers were entered concurrently into an integrated database of the statistic software SPSS by the interviewer who was in front of a computer with a headset while doing the interview (CATI). The research assistant supervisor checked five percent of the data collected against the voice recording, providing feedbacks to each interviewer regarding errors, way of going in depth in a specific question, and checking that appropriate corrections were performed in the database. This data quality checking was performed concurrently as the interviews were being performed.

In addition, other four distinct research assistants independently abstracted the corresponding clinical information from medical records using standardized forms after the telephone interview was achieved. They were also unaware of the condition of severe maternal morbidity of each woman prior data collection. Five percent of medical records were also abstracted twice as a quality control procedure performed by another supervisor, with the necessary corrections being performed as required. This information was then introduced in another SPSS database. After testing for consistency and cleaned, both databases were matched by the hospital register number and then merged.

For the analysis of construct validity, we performed the Cronbach's Alpha statistical analysis to verify the internal consistency. Considering this questionnaire was designed to get information on severe maternal morbidity and not aimed to establish any score graduation, we adopted the criterion of Bowling [16], accepting values of Cronbach's alpha coefficient equal to or greater than 0.5. The correlation between pairs of items from the questionnaire referring to diagnosis or procedures for severe maternal morbidity was performed using Spearman's correlation coefficients.

The capacity of the questionnaire to identify women with severe maternal morbidity was assessed using the information from medical records as the "gold standard". Sensitivity and specificity were calculated for each question and combinations of questions for the main diagnosis and procedures related to the topic under study. In addition, we calculated likelihood ratios for the performance of each question and they were our main estimators of accuracy. Likelihood ratios were calculated as sensitivity/(1-specificity). A likelihood ratio > 10 was considered highly correlated with confirming the event recalled by the woman with the medical records [13,17]. The 95% confidence intervals (Fleiss' quadratic for sensitivity and specificity and classic Wald for likelihood ratio) and p-values for characteristics comparison were also calculated. All statistical analyses were performed using the SPSS v.11.5 and Epi.Info v.6.04d softwares.

## Results

**Figure 1:**
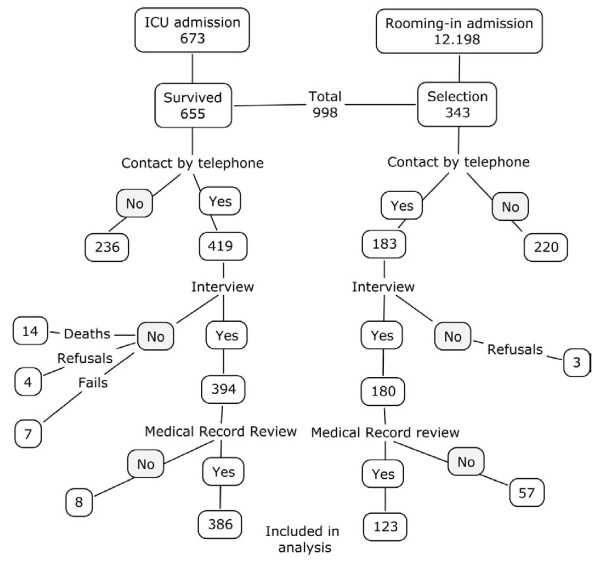
**Flowchart for selection of cases and controls for the validation study**.

During the period, 673 women were admitted to the obstetric ICU, with 655 survivors. In the same period, 12,198 women were admitted to the rooming-in ward with no severe maternal morbidity as defined above. Among them, a list of 343 has been selected for telephone interview. Initially we decided to select a proportion of 2 cases for 1 control, presuming that there would be more difficult to trace controls than cases in order to achieve the desired proportion of 3 cases to 1 control. Therefore there were initially 998 eligible women for the study. A total of 602 women were reached through telephone (60.3% of total success rate in reaching contact) and 574 were interviewed, 394 women with severe complications and 180 without (60% success rate in interviewing among cases and 52% among controls). Of the reached women with no interview, seven women refused to participate in the study (3 controls and 4 cases), fourteen were dead at the moment of the interview (cases) and in seven cases it was not possible to perform the interview after the contact (Figure 1).

Of women interviewed, 386 from ICU and 123 from the rooming-in ward had also their clinical records carefully checked for an event of severe maternal morbidity. In eight cases the medical records were unable to be traced at the time of the study. In order to keep a proportion of around 3:1 (cases:controls), the control's medical records review was stopped as soon as this proportion was achieved; therefore, 57 controls did not have their medical records reviewed. The age, parity and marital status are shown in Table 1. Additional analysis was performed to compare the characteristics of women with severe maternal morbidity who were and who were not interviewed and no significant differences were found in age, parity, marital status and mode of delivery (data not shown).

**Table 1:** Distribution of women according to some characteristics by the occurrence of severe maternal morbidity (SMM)

Characteristic	SMM	No SMM	**p**^**&**^
	%	%	
**Age at birth (years)**			0.030
Up to 19	10.4	13.0	
20 - 24	22.5	34.1	
25 - 29	26.7	22.8	
30 - 34	21.8	20.3	
≥ 35	18.7	9.8	
			
**Parity **#			0.119
0	42.1	33.9	
1	26.6	36.5	
≥ 2	31.4	29.6	
			
**Marital status **^@^			0.519^$^
With partner	68.0	71.7	
Without partner	32.0	28.3	
			
**Total**	386	123	

*SMM cases: with at least one related diagnosis and/or procedure

^&^Pearson Chi-square test; ^$ ^Chi-square test with Yates correction

Missing information for: ^@ ^11 women; # 104 women

The frequency of obstetrical complications and indicators of management as recorded in the medical records are presented in Table 2 for women who experienced a severe maternal morbidity during pregnancy or childbirth. There were no cases of abortion contributing to this morbidity. The internal consistency is also shown on Table 2. The Cronbach's alpha was obtained using the total of women answering the questionnaire (cases and controls in a total of 509 subjects). The general Cronbach's Alpha was 0.655, considered acceptable by the chosen criteria. The individual values show what would be the changes in general Cronbach's Alpha if each item were excluded from the analysis.

**Table 2:** Distribution of women who had an episode of severe maternal morbidity according to main diagnosis or procedures performed and internal consistency through Cronbach's alpha for each item of the questionnaire

	Main diagnosis or procedure	Cronbach's Alpha
**Diagnosis**	%	
Eclampsia	7.3	0.659
Hemorrhage before, during or after delivery	16.3	0.626
Sepsis	6.7	0.661
Jaundice	1.8	0.667
**Procedures**		
Admission to ICU	99.5	0.600
Use of mechanical ventilation	17.1	0.622
Transfer to other hospital	28.8	0.658
Laparotomy	6.7	0.649
Hysterectomy	7.5	0.645
Post partum stay above 7 days	29.5	0.621
Blood transfusion	25.4	0.601
		**General 0.655**

**Total women**	**386**	**509**

The correlation between items is shown in Table 3. In general, the diagnostic items were poorly correlated in between them; there were direct correlations between infection and the others diagnoses and between eclampsia and jaundice. Assessing the correlation between diagnostic criteria and procedures, infection and jaundice were poorly correlated with them; however, eclampsia and hemorrhage were correlated with most procedures. Among the procedures, there were statistical significant correlation between all pairs, except for inter-hospital transfer and laparotomy and inter-hospital transfer and hysterectomy.

**Table 3:** Spearman's correlation coefficient (r) between pairs of items from the questionnaire referring to diagnosis or procedures for severe maternal morbidity (n = 509)

	Eclampsia	Hemorrhage	Infection	Jaundice	Admission to ICU	Mechanical ventilation	Inter-hospital transfer	Laparotomy	Hysterectomy	Postpartum stay > 1 week	Blood transfusion
Eclampsia	1.000	0.060	**0.102**	**0.096**	**0.134**	**0.107**	**0.093**	0.007	-0.066	**0.131**	**0.089**

Hemorrhage	0.060	1.000	**0.144**	0.065	**0.169**	**0.166**	0.058	**0.130**	**0.322**	**0.186**	**0.656**

Infection	**0.102**	**0.144**	1.000	**0.094**	0.059	**0.107**	0.017	0.081	0.076	0.028	**0.130**

Jaundice	**0.096**	0.065	**0.094**	1.000	**0.091**	**0.097**	0.042	0.074	-0.051	0.073	**0.133**

Admission to ICU	**0.134**	**0.169**	0.059	**0.091**	1.000	**0.342**	**0.329**	**0.169**	**0.150**	**0.405**	**0.279**

Mechanical ventilation	**0.107**	**0.166**	**0.107**	**0.097**	**0.342**	1.000	**0.171**	**0.135**	**0.200**	**0.261**	**0.238**

Inter-hospital transfer	**0.093**	0.058	0.017	0.042	**0.329**	**0.171**	1.000	0.034	0.017	**0.188**	**0.093**

Laparotomy	0.007	**0.130**	0.081	0.074	**0.169**	**0.135**	0.034	1.000	**0.311**	**0.134**	**0.208**

Hysterectomy	-0.066	**0.322**	0.076	-0.051	**0.150**	**0.200**	0.017	**0.311**	1.000	**0.125**	**0.349**

Postpartum stay > 1 week	**0.131**	**0.186**	0.028	0.073	**0.405**	**0.261**	**0.188**	**0.134**	**0.125**	1.000	**0.258**

Blood transfusion	**0.089**	**0.656**	**0.130**	**0.133**	**0.279**	**0.238**	**0.093**	**0.208**	**0.349**	**0.258**	1.000

Bold means p < 0,05

Table 4 shows the performance of the questionnaire for severe morbidity by questions and combinations of questions against medical records. Overall, women did not recall accurately the occurrence of obstetric complications, especially hemorrhage, infection and jaundice. The likelihood ratios were < 5 for them, while for eclampsia were nearer 10. However, none of questions combination related to diagnoses reached a LR above 10.

**Table 4:** Performance of questioning women in a survey for the diagnosis of eclampsia, hemorrhage, infection, jaundice and several procedures during pregnancy or childbirth as indicators of SMM (n = 509)

	Sensitivity [95% CI]	Specificity [95% CI]	Likelihood ratio [95% CI]
**Eclampsia**			
Woman had convulsions, seizures or "crisis" during pregnancy, delivery or postpartum	96.4 [79.8-99.8]	87.5 [84.2 - 90.3]	7.7 [6.4-9.9]
The previous plus: woman had not had convulsions before	89.3 [70.6-97.2]	90.9 [87,8 - 93,2]	9.8 [7.16-13.30]
The previous plus with an increase in blood pressure, swelling and "turbid vision" during pregnancy, delivery or postpartum	35,7 [19,3-55,9]	95,4 [93,0 - 97,0]	7.8 [4.10-14.85]
**Hemorrhage**			
Women had bleeding during pregnancy or an increased bleeding during delivery or postpartum	81.0 [68.7-89.4]	69.7 [65.2 - 73.9]	2.7 [2.22-3.22]
The previous plus: bleeding wet the clothes, the bed or the floor	55.6 [42.6-67.9]	81.6 [77.6 - 85.0]	3.0 [2.25-4.06]
**Infection**			
Woman had high fever during pregnancy or after delivery	69.2 [48.1-84.9]	77.2 [73.2 - 80.8]	3.0 [2.24-4.12]
The previous plus chills, with no other disease, with stinky vaginal discharge	23.1 [9.8-44.1]	92.8 [90.0 - 94.8]	3.2 [1.47-6.88]
**Jaundice**			
Women became yellow during pregnancy or after birth	57.1 [20.2 - 88.2]	82.3 [78.6 - 85.5]	3.2 [1.65 - 6.29]
The previous plus: Nobody else in the family or neighborhood became yellow next the time she also became yellow	57.1 [20.2 - 88.2]	83.3 [79.6 - 86.4]	3.4 [1.75 - 6.68]
**Procedure**			
Hysterectomy	100.0 [85.4 - 100.0]	99.6 [98.3 - 99.9]	240.0* [60.20-956.88]
Admission to Intensive Care Unit	97.1 [94.8 - 98.5]	96.0 [90.4 - 98.5]	24.3* [10.29-57.33]
Blood transfusion	89.8 [81.6 - 94.7]	92.9 [89.9 - 95.1]	12.6* [8.90-18.19]
Laparotomy	69.2 [48.1 - 84.9]	88.2 [84.9 - 90.9]	5.9 [4.12-8.36]
Inter-hospital transfer	86.5 [78.4 - 92.0]	74.6 [70.0 - 78.8]	3.4 [2.84-4.10]
Mechanical ventilation	84.8 [73.4 - 92.1]	70.9 [66.4 - 75.0]	2.9 [2.44-3.48]
Postpartum stay > one week	87.7 [79.9 - 92.9]	65.3 [60.4 - 70.0]	2.5 [2.17-2.94]

Information recalled by women on hysterectomy, ICU admission and blood transfusion were found to be highly correlated with the correspondent events in medical records (LR from 12.7-240). However, laparotomy, inter-hospital transfer, mechanical ventilation and postpartum stay above one week performed worse than those, with LR well below 5. For the procedures with the best performance an additional analysis was performed to try to identify factors possibly associated with this finding. The only factor that showed to be significant was the time elapsed between the occurrence of the morbidity episode and the interview. The higher length of time was associated with poorer recall (data not shown in table).

## Discussion

This study addressed the question of how accurately women recall events occurred during their pregnancy and childbirth, especially those related to severe complications that could be life threatening. The questionnaire developed for this purpose has shown to have an acceptable internal consistency. Although there is no ideal cutoff point for the design of an indicator, considering the complexity of the phenomenon that was intended to measure, and that this questionnaire was built for getting information on severe maternal morbidity and not to establish any score graduation, the value obtained could be considered as acceptable for internal consistency [16]. The correlation analysis also showed a proper correlation between procedures and diagnostic criteria and in-between procedures supported by the clinical status of the patients.

Process indicators worked better as indicators of severe maternal morbidity when recalled by women than obstetric complications. Hysterectomy, ICU admission and blood transfusion were accurately recalled. Eclampsia could be regarded as in an upper intermediate accuracy level, while hemorrhage, infection and jaundice were poorly recalled. We also found that inaccuracy was associated with increasing time between the delivery and the interview.

Obstetric complications are commonly assumed as among the most remarkable events that a woman can experience during pregnancy and childbirth. However, previous studies have shown that women recall obstetric complications in a varied way. Most of these studies found that eclampsia could be satisfactorily recalled, while there was more uncertainty regarding hemorrhage, dystocia and infection [4,6]. In fact, our study showed that eclampsia was the only obstetric complication that nearly achieved a reliable accuracy.

The length of time from pregnancy until interview is one of the factors that may affect the way that women recount their stories of pregnancy and childbirth, which is documented in literature as well. Although one study [18] suggests that long-term maternal recall (thirty or more years) is both reproducible and accurate for many factors related to pregnancy and delivery including pregnancy complications, some authors show a correlation between length of time elapsed since the pregnancy and poor recall of some pregnancy related complications such as anemia, high blood pressure [19] and the report of gestational age [20].

There may be possible explanations for this observation since severely ill women during pregnancy and childbirth may experience altered mental states that transiently impair their memories about the events associated with the complication. Amnesia and memory gaps are described as frequent components of severe maternal morbidity [21,22]. Apart from the time itself, these factors can contribute to a low performance of questions referring to obstetric complications in surveys.

On the other hand, process indicators have been used as proxies of severe maternal morbidities during the last 15 years [15]. Notwithstanding Joffe and Grisso [23] reported disagreement in areas involving technical knowledge or intervention between medical records and maternal interview, we found that some of them, especially ICU admission, hysterectomy and blood transfusion were recalled with high accuracy. These three indicators are consistently associated with severe maternal morbidity in several studies [24]. However they had never been tested before in population surveys.

Current findings encourage the use of these process indicators and similar questionnaires in population studies, like a DHS, as an adjunct to improve our understanding of maternal health. Besides some concerns that may have regarding using process indicators worldwide for this purpose, considering the inequalities in accessibility of these procedures for all women during childbirth could introduce a bias, they were consistently found to be correctly recorded by women experiencing a severe maternal morbidity episode, as found in the database for Latin America from the WHO Global Survey on maternal and perinatal health [24].

Nevertheless, there are some points that should be addressed. Once medical records were our gold standard, our validation study relies only on the recall of hospitalized women. This could be an unavoidable selection bias. In places where healthcare services are irregular or insufficient, women attending them may be different from those who do not attend. In Brazil, and particularly in the region where the study was conducted, the rate of hospitalization for delivery is very high (> 98%) and it may have minimized this bias. Other possible selection bias is that we only included women that could be reached by telephone. Although there were no significant differences in the characteristics of women with severe maternal morbidity who were and who were not interviewed regarding age, parity, marital status and mode of delivery, women who no longer have telephone or who have changed their phone number could possibly be different from those who were interviewed. We had to base our study in telephone interviews considering the feasibility and the practical aspects of this approach. However, in this region the telephone coverage is very high, mainly when we consider the sum of fixed lines and mobile phones (above 76%) [25]. In fact, all women who were eligible to this study were able to give any phone number at the moment of her hospital admission for future contact. However, we were unable to estimate the actual impact of this selection bias in the study.

In addition, severe maternal morbidity is a condition with low prevalence in general obstetric populations, and there is an interaction between the accuracy, the prevalence of self-reported morbidity and the actual prevalence of morbidity. The occurrence of high specificities associated with low actual prevalence results in accuracy being essentially determined by the specificity. The use of accuracy as the main indicator of validity can eventually overestimate the validity [26]. In our study, most of the questions and combinations tested revealed higher specificities and we artificially increased the prevalence of complications by including only 123 normal controls. For that reason, some authors suggest that the inclusion of healthy controls is likely to lower the occurrence of false-positive results, thereby increasing specificity [27].

Caution should be exercised when applying these findings to general populations, although our main estimator of accuracy, the likelihood ratio, is less dependent of the base rate of the target event [13]. This should be a matter of concern when using the data from the validation of these questions for the results of the recent Brazilian DHS which used some of the questions already validated [7].

Although there is no consensus about maternal self reports in providing a valid estimate of the prevalence of obstetrical complications [9], we conclude that it is possible to assess severe maternal morbidity through population surveys. We observed that process indicators are more accurately recalled than obstetric complication per se and that length of time from pregnancy until interview can affect the maternal recall. In this context, we would recommend the addition of locally relevant process indicators in the questionnaires. Finally, population surveys as the DHS may be useful as an exploratory tool where more precise and elaborated approaches are not feasible. Furthermore, additional research is needed on the determinants of poor accuracy and the use of process indicators in population surveys.

## Disclosure of interests

The authors report no conflict of interests. The authors alone are responsible for the content and writing of the paper.

## Authors' contributions

JPS and JGC had the original idea for the study. JPS wrote the first version of the proposal with orientation from JGC and MAP. JGC got the grant for implementation of the study. JPS, TMG were responsible for data collection. MHS, JPS, JGC and RCP were responsible for data analysis. JPS, RCP and RSC wrote the first draft of the paper and then all the others gave important inputs and suggestions for interpretation and improvement of the manuscript. All authors have read the final version of the article and agreed with it.

## Supplementary Material

Additional file 1 **Questionnaire on severe maternal morbidity**. Click here for file
