# Supplementary material for: Development and validation of a questionnaire to identify severe maternal morbidity in epidemiological surveys
Source: Reprod Health. 2010 Jul 21;7:16. doi: 10.1186/1742-4755-7-16 (PMC2915965; doi:10.1186/1742-4755-7-16)
Supplement: Additional file 1 — Questionnaire on severe maternal morbidity. [file 1742-4755-7-16-S1.DOC]

| Questionnaire on severe maternal morbidity |
| --- |
|  |
| **Identification** Date of Interview: ____/____/_____  A) Register number in the Study: |__|__| B) Initials: |__|__|__|__|__|__| C) Age: |__|__|  D) What was the last level that you attended at school? ________________________ |
|  |
| 1) We are talking about your pregnancy that finished in ______ (year when the delivery or abortion occurred). During that pregnancy, when you were admitted at the hospital, did you have any kind of complication?   Yes  No (go to question 3) |
| 2) What is the name of this complication you had?  ___________________________________ |
| 3) In what date this delivery or abortion occurred?  |__|__| / |__|__| / |__|__| (dd/mm/yy) |
| **Module eclampsia** |
| 4) Did you have seizures, "crises" or "attacks" during pregnancy, childbirth or after childbirth?    Yes  No (go to question 5) |
| 4.1) Have you ever had seizures before?  Yes  No |
| 4.2) Do you know if you have increased blood pressure during pregnancy?   Yes  No |
| 4.3) Did you have swelling in the legs, face or hands during pregnancy, childbirth or after childbirth?  Yes  No |
| 4.4) Did you have blurred vision or blurring of sight during pregnancy, childbirth or after childbirth?  Yes  No |
| **Module hemorrhage** |
| 5) Did you have bleeding during pregnancy, or an increased bleeding during delivery or after childbirth?   Yes  No (go to question 6) |
| 5.1) This bleeding wet your clothes, the bed or the floor?  Yes  No |
| 5.2) The bleeding started before the birth of the child?  Yes  No |
| 5.3) If the bleeding started before birth, did you feel pain while displaying the bleeding?  Yes  No |
| 5.4) If not, did you have other episodes of bleeding during pregnancy?  Yes  No |
| Module sepsis |
| 6) Did you have a high fever during pregnancy or after childbirth?  Yes No (go to question 7) |
| 6.1) This came with fever chills?  Yes No |
| 6.2) Have you been sick with some other disease during pregnancy?  Yes No |
| 6.3) Did you have a very smelly discharge?  Yes No |
| Module jaundice |
| 7) Did you turn yellow during pregnancy or after childbirth?  Yes No (go to question 8) |
| 7.1) Were you yellow at birth?  Yes No |
| 7.2) Did you turned yellow after childbirth?  Yes No |
| 7.3) Does anyone else in your family or neighborhood turned yellow around the time when you also have been?  Yes No |
| Module of generic indicators of severity |
| 8) Were you admitted to the ICU at this time?  Yes No |
| 9) Did you need breathing apparatus, on this occasion?  Yes No |
| 10) Did you have to be transferred from one hospital with fewer resources because of this complication?  Yes No |
| 11) Did you have to be operated by the abdomen, excluding CS?  Yes No |
| 12) Did your uterus have to be withdrawn on this occasion?  Yes No |
| 13) After delivery, did you stay more than a week in the hospital?  Yes No |
| 14) Did you receive a blood transfusion, on this occasion?  Yes No |
| 15) How you consider your health status today compared to before the occurrence of complications :  Worst Best Same |
